# Urine creatine metabolite panel as a screening test in neurodevelopmental disorders

**DOI:** 10.1186/s13023-020-01617-z

**Published:** 2020-12-02

**Authors:** Shalini Bahl, Dawn Cordeiro, Lauren MacNeil, Andreas Schulze, Saadet Mercimek-Andrews

**Affiliations:** 1 Division of Clinical and Metabolic Genetics, The Hospital for Sick Children, 555 University Avenue, Toronto, ON M5G 1X8 USA; 2 Metabolic Laboratory, Department of Laboratory Medicine, The Hospital for Sick Children, Toronto, ON USA; 3 Department of Medical Genetics, University of Alberta, Edmonton, AB USA; 4 Genetics and Genome Biology Program, Research Institute, The Hospital for Sick Children, Toronto, ON USA; 5 Department of Pediatrics, University of Toronto, Toronto, ON USA

**Keywords:** Cerebral creatine deficiency syndromes, Creatine, Guanidinoacetate, Global developmental delay, Epilepsy

## Abstract

**Background:**

Cerebral creatine deficiency disorders (CCDD) are inherited metabolic disorders of creatine synthesis and transport. Urine creatine metabolite panel is helpful to identify these disorders.

**Methods:**

We reviewed electronic patient charts for all patients that underwent urine creatine metabolite panel testing in the metabolic laboratory at our institution.

**Results:**

There were 498 tests conducted on 413 patients. Clinical, molecular genetics and neuroimaging features were available in 318 patients. Two new patients were diagnosed with creatine transporter deficiency: one female and one male, both had markedly elevated urine creatine. Urine creatine metabolite panel was also used as a monitoring test in our metabolic laboratory. Diagnostic yield of urine creatine metabolite panel was 0.67% (2/297). There were six known patients with creatine transporter deficiency. The prevalence of creatine transporter deficiency was 2.64% in our study in patients with neurodevelopmental disorders who underwent screening or monitoring of CCDS at our institution.

**Conclusion:**

Even though the diagnostic yield of urine creatine metabolite panel is low, it can successfully detect CCDD patients, despite many neurodevelopmental disorders are not a result of CCDD. To the best of our knowledge, this study is the first Canadian study to report diagnostic yield of urine creatine metabolite panel for CCDD from a single center.

## Introduction

Cerebral creatine deficiency disorders (CCDD) are inherited metabolic disorders of creatine biosynthesis and transport. Two enzymes, arginine-glycine amidino transferase (AGAT) (EC 2.1.4.1) [encoded by *GATM* (MIM#602360)] and guanidinoacetate methyltransferase (GAMT) (EC#2.1.1.2) [encoded by *GAMT* (MIM#602360)] are required for the stepwise biosynthesis of creatine. Both enzyme deficiencies, AGAT (MIM#612718) and GAMT (MIM#612736) are inherited autosomal recessively. Circulating creatine is taken up by a sodium-dependent membrane bound creatine transporter (CRTR) [encoded by *SLC6A8* (MIM#300036)] into high energy demanding tissues such as brain and muscle. CRTR deficiency (MIM#300352) results in an X-linked disorder. There have been < 120 patients with GAMT deficiency, < 20 patients with AGAT deficiency and < 200 patients with CRTR deficiency reported in the literature [1].

Clinical features of all three CCDD include global developmental delay, intellectual disability, seizures, movement disorder and behavioral problems, which are not specific to CCDD alone. However, the characteristic biochemical features of each CCDD is unique: elevated guanidinoacetate in body fluids in GAMT deficiency, low or low normal urine guanidinoacetate and low plasma guanidinoacetate in AGAT deficiency and elevated urine creatine in CRTR deficiency in males. Because of these abnormalities, a urine creatine metabolite panel that measures guanidinoacetate, creatine and creatinine can be used as a screening test for CCDD. As all three disorders impair creatine metabolism and transport, males and females with GAMT and AGAT deficiencies and males with CRTR deficiency have absent creatine in brain tissue when measured by magnetic resonance spectroscopy (MRS). Abnormal urine creatine metabolite panels and abnormal brain MRS guide the molecular genetic confirmation by direct sequencing of *GAMT*, *GATM* and *SLC6A8*. Females with a heterozygous likely pathogenic variant in *SLC6A8* can have normal urine creatine or creatine peak in MRS [1]*.* Identification of patients or individuals in the neonatal or early infantile period and initiation of the treatment results in a normal neurodevelopmental outcome in GAMT and AGAT deficiencies [2–4], which necessitates early identification of these disorders.

We performed a retrospective review of a urine creatine metabolite panel that measures guanidinoacetate, and creatine to determine (1) The diagnostic yield of the urine creatine metabolite panel when used as a screening for CCDD; (2) Prevalence of CCDD in the study population and at our institution; and (3) Diagnosis of secondary cerebral creatine deficiency disorders using the urine creatine metabolite panel.

## Material and methods

The Hospital for Sick Children’s Research Ethics Board approved this study (Approval#1000063421). All individuals who underwent a urine creatine metabolite panel in our Metabolic Diseases Laboratory between January 2013 and February 2019 were included in our study. Urine samples were sent from pediatrics, developmental pediatrics, neurology, clinical genetics, and metabolic clinics at our institution or from outside of our institution for diagnostic investigations. Our Metabolic Diseases Laboratory is one of the clinical reference laboratories for the measurement of urine guanidinoacetate and creatine in Canada.

All measurements were performed at the time of send-out to the clinical biochemical laboratory as part of the clinical diagnostics. At the time of study start date (February 2019), we acquired all urine creatine metabolite panel results performed in the Metabolic Diseases Laboratory according to the methods and standard operating procedures of this laboratory. All results were interpreted by a clinical or biochemical geneticist, who are experts in the inconclusive range between the normal reference interval and clinically relevant abnormal measurements.

We entered the clinical features, biochemical investigations and neuroimaging results of all individuals who had medical records at our institution into an Excel Database.

Concentrations of urine guanidinoacetate, and creatine were determined using an underivatized liquid chromatography tandem mass spectrometry (LC–MS/MS) protocol using a previously reported method [5]. Briefly, random urine collections from patients were aliquoted and stored at − 20 °C until testing. Once thawed and vortexed, 5 µl of patient sample was added to a diluent containing stable isotope internal standards for each analyte. Specimens were run in concert with calibrators and quality control materials on AB SCIEX API4000 QTrap and Waters Xevo TQ-S Mass Spectrometers using a Waters Atlantis column (dC18 150 × 3.0 mm, 3 µm). Comparison testing was performed at the time of the method development and validation and confirmed that there was no bias between the two instruments. Testing proficiency for creatine metabolite measurement was routinely assessed, meeting both internal (%CV < 10) and external quality program (ERNDIM) acceptance criteria. Creatinine was measured using the alkaline picrate method. Briefly, picric acid in an alkaline medium reacts with creatinine to form an orange coloured complex. Intensity of the coloured complex is measured by automated chemistry analyzer (Abbot Architect immunoassay analyzer), which is directly proportional to the concentration of creatinine in a urine sample. Guanidinoacetate, and creatine concentrations were normalized to creatinine.

Fisher’s exact test was used to compare clinical and neuroimaging features of CCD patients with patients with no molecular genetic diagnosis of CCD. Kruskal–Wallis rank sum test was used to identify if there were significant sex differences within each reference age group. All the statistical analysis was conducted using R software (version 1.2.1335). A *p* value of < 0.05 was considered to be statistically significant.

## Results

**Fig. 1:**
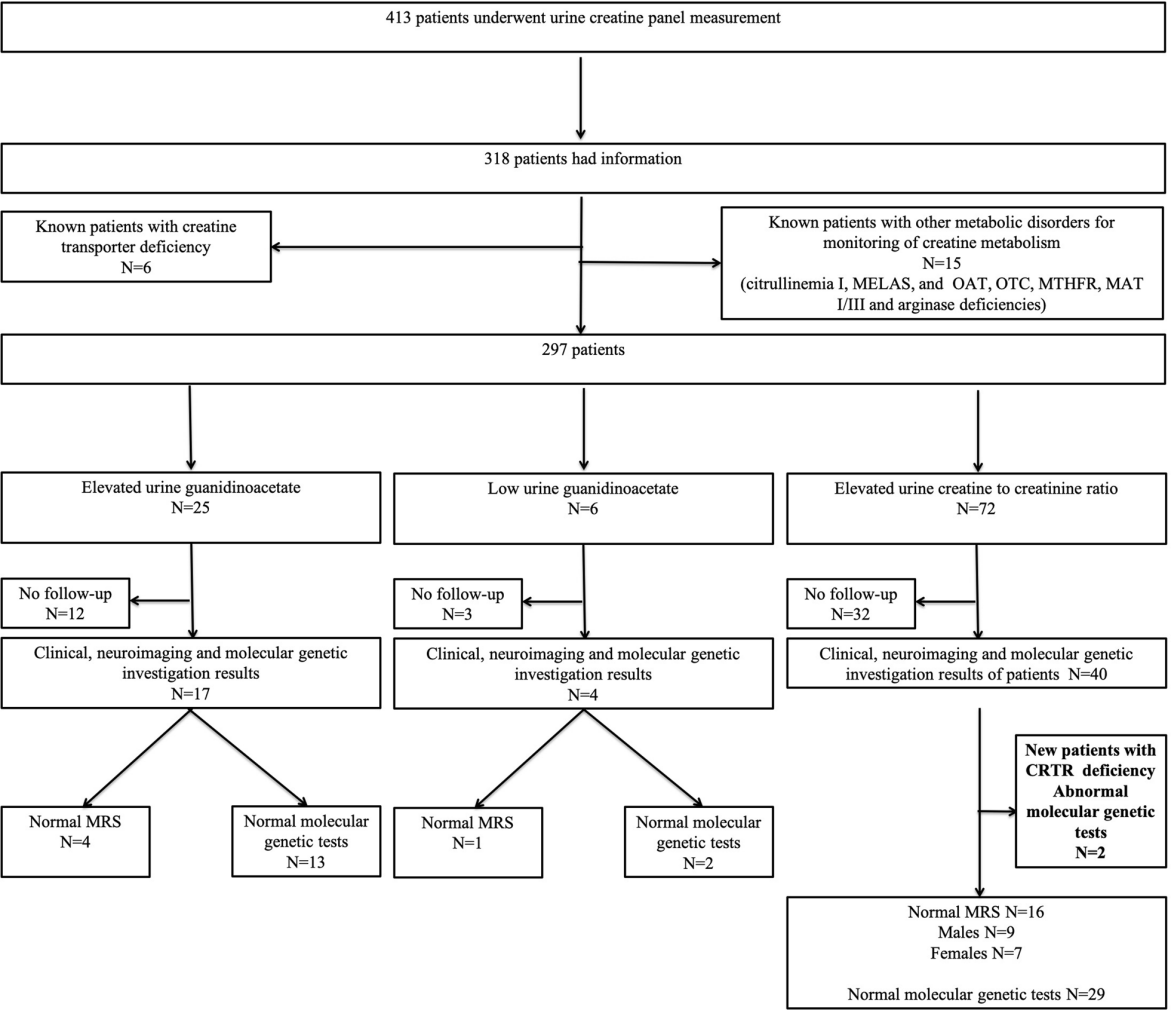
Number of abnormal urine creatine panel and their follow-up investigations are depicted in Fig. 1

**Fig. 2:**
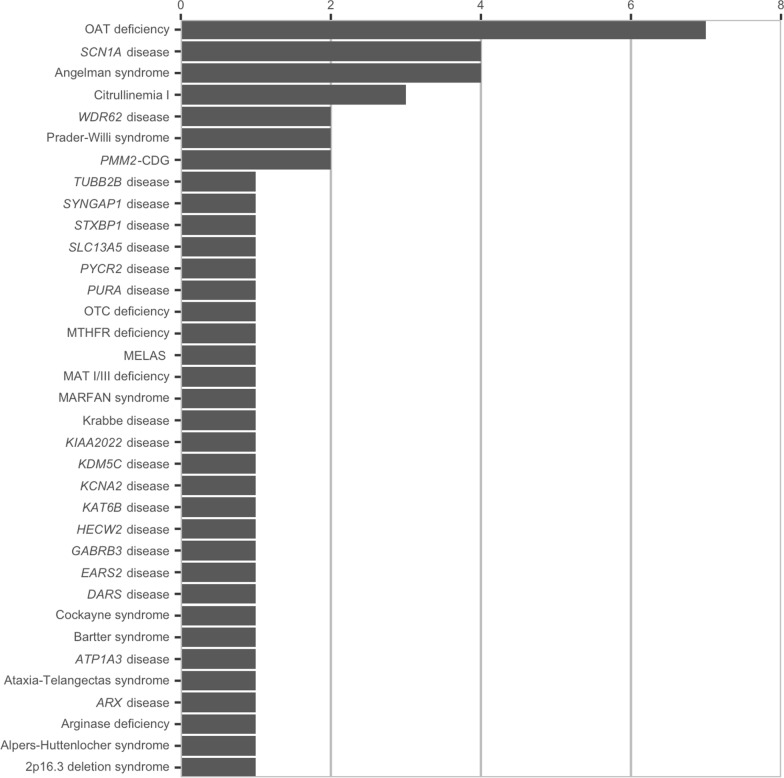
Number of patients and their genetic diagnoses are depicted in Fig. 2

There were 498 urine samples for the measurement of urine creatine metabolite panel in 413 patients. Ninety-five patients, including 8 who were > 19 years old (age range was 19.5–57.5 years), were excluded as there was no clinical information provided. We had clinical, molecular genetics and neuroimaging features for 318 of the 413 patients with urine creatine panel metabolite measurements. Twenty-one out of 318 patients had urine creatine metabolite panel measurement for monitoring of creatine metabolism for their known inherited metabolic disorders including six patients with creatine transporter deficiency [6] and 15 patients with seven different inherited metabolic disorders (ornithine-δ-aminotransferase deficiency, ornithine transcarbamylase deficiency, citrullinemia type I, arginase deficiency, MELAS, MTHFR deficiency, MAT I/III deficiency) (Fig. 1 and 2, Additional file 1). We excluded those patients from our data analysis. The remaining 297 patients and their urine creatine panel metabolite measurements were analyzed. There were 112 females and 185 males. We depicted the number of patients with an abnormal urine guanidinoacetate and urine creatine in Fig. 1. We summarized the average, median, percentiles and their numbers for guanidinoacetate and creatine levels based on the age dependent reference ranges for males and females in Table 1. Urine guanidinoacetate (*p* = 0.001856) and creatine (*p* = 0.03946) levels were significantly different between males and females for the age group 0–60 months, whereas in other age groups, there was no statistically significant difference between males and females (Table 1). The average creatinine level was 6442 ± 5093.29 SD μmol/L (range 65–35,658).

**Table 1:** Urine creatine panel metabolites and their age and sex related distribution

Urine creatine panel metabolites	Age dependent reference range	Numbers	Female	Male	Statistical analysis (non-parametric Kruskal–Wallis rank sum test)
			Number	Number	
			Mean ± SD	Mean ± SD	
			Range	Range	
Urine guanidinoacetate	0–60 months (Reference range 5–150 mmol/mol creatinine)	Number	58	109	*p* = 0.001856*
		Average ± SD	104.0 ± 51.9	81.81 ± 44.3	
		Median	100	71	
		Range	15.0–250.0	9.00–281.00	
		2.5th percentile	25	26	
		25th percentile	61	54.25	
		75th percentile	138.5	93.75	
		97.5th percentile	204.9	195.5	
	60–192 months (reference range 15–130 mmol/mol creatinine)	Number	48	67	*p* = 0.569
		Average ± SD	64.25 ± 39.6	69.21 ± 87.8	
		Median	54	54	
		Range	8–171	1–573	
		2.5th percentile	10.275	10.625	
		25th percentile	37	34.25	
		75th percentile	85.7	78.5	
		97.5th percentile	151.625	237.025	
	192–228 months (reference range 11–60 mmol/mol creatinine)	Number	6	9	*p* = 0.07825
		Average ± SD	60.86 ± 29.1	34.90 ± 15.8	
		Median	44	34	
		Range	29–97	13–65	
		2.5th percentile	30.35	13.9	
		25th percentile	39.5	24.5	
		75th percentile	88.5	42.75	
		97.5th percentile	96.4	61.4	
Urine creatine	0–60 months (reference range 14–830 mmol/mol creatinine)	Number	58	109	*p* = 0.03946*
		Average ± SD	777.3 ± 490.0	645.6 ± 537.0	
		Median	720	533.5	
		Range	14.0–2333.0	4.0–3915.0	
		2.5th percentile	31.85	13.925	
		25th percentile	383	327.5	
		75th percentile	1059	885.2	
		97.5th percentile	1710.75	1844.43	
	60–192 months (reference range 16–649 mmol/mol creatinine)	Number	48	67	*p* = 0.8339
		Average ± SD	424.83 ± 1217.0	290.8 ± 471.0	
		Median	153	161	
		Range	3.00–8756.00	3.0–3518.0	
		2.5th percentile	5.825	4	
		25th percentile	25.25	28	
		75th percentile	398.25	389	
		97.5th percentile	1080.78	907.075	
	192–228 months	Number	6	9	*p* = 0.6594
	(reference range 19–173 mmol/mol creatinine)	Average ± SD	47.0 ± 64.6	68.6 ± 164.0	
		Median	17	11	
		Range	5.0–186.0	5.0–531.0	
		2.5th percentile	5.4	5.225	
		25th percentile	9	8	
		75th percentile	51.5	16.5	
		97.5th percentile	167.1	428.625	

Urine creatine panel metabolites and their age and sex related distribution in 297 patients who underwent urine creatine panel measurement for the diagnostic investigations are listed

Two patients had a new diagnosis of CRTR deficiency during the study period: one female with a next generation sequencing panel for intellectual disability and elevated urine creatine, and one male by whole exome sequencing (WES) and elevated urine creatine. We summarized both patients in Table 2.

**Table 2:** Two new patients, who had diagnosis of CRTR deficiency during the study period are summarized in Table 2 for their clinical features and genotypes

Diagnosis current age/ sex	Age of onset/age of diagnosis	Clinical features	Urine creatine panel	Brain MRI/ MRS (age)	Molecular genetic test results
1/CRTR deficiency/6yrs/F	6mo/3yrs	GDD, hypotonia, seizures, ASD, aggressive behavior	↑ Creatine (4118 mmol/mol creatinine; reference range 14–830)	N/decreased creatine (3yrs)	HTZ de novo c.1583del (p.Pro528Argfs*67) in *SLC6A8* (next generation sequencing panel for ID)
			N guanidinoacetate (36.6 mmol/mol creatinine; reference range 5–150)		
2/CRTR deficiency/5yrs/M	1 yr/2.5yrs	GDD, febrile seizure	↑ Creatine (3915 mmol/mol creatinine; reference range 14–830)	NP	Hemizygous c.321_323delCTT (p.Phe107del) in *SLC6A8* (WES) (mosaic mother 10/163 reads in the mother, asymptomatic)
			N guanidinoacetate (42 mmol/mol creatinine; reference range 5–150)		

ASD = autism spectrum disorder; CRTR = creatine transporter; GDD = global developmental delay; HTZ = heterozygous; ID = intellectual disability; MRI = magnetic resonance imaging; MRS = magnetic resonance spectroscopy; N = normal; NP = not performed; WES = whole exome sequencing

Elevated guanidinoacetate was identified in 8.42% (25/297) of the patients and GAMT deficiency was excluded in 52% (13/25) of those patients either by normal brain MRS or by normal molecular genetic investigations or both (Table 3). We depicted elevated urine guanidinoacetate levels for 13 patients who had normal brain MRS, molecular genetic investigations or both in Additional file 2.

**Table 3:** Abnormal urine guanidinoacetate levels and their follow-up investigations

Age groups and reference ranges	Elevated guanidinoacetate	Low guanidinoacetate
	Number (range)	Number (range)
	Follow-up investigations	Follow-up investigations
0–60 months (reference range 5–150 mmol/mol creatinine)	N = 15	N = 0
	(range = 158.0–281.0; median 180.0)	
	Normal genetic investigations for GAMT deficiency n = 5	
	Normal MRS and normal genetic investigations for GAMT deficiency n = 2	
	No confirmatory follow-up investigations n = 8	
60–192 months (reference range 15–130 mmol/mol creatinine)	N = 7	N = 6
	(range = 132.0–573.0; median 153.0)	(range = 1.000–12.000; median 9)
	Normal MRS n = 2	Normal MRS n = 1
	Normal genetic investigations for GAMT deficiency n = 3	Normal genetic investigations for AGAT deficiency n = 2
	No confirmatory follow-up investigations n = 2	No confirmatory follow-up investigations n = 3
192–228 months (reference range 11–60 mmol/mol creatinine)	N = 3	N = 0
	(range = 65.00–97.00; median 88.5)	
	Normal genetic investigations for GAMT deficiency n = 1	
	No confirmatory follow-up investigations n = 2	

Low guanidinoacetate was identified in 2.02% (6/297) of the patients and AGAT deficiency was excluded in 50% (3/6) of those patients either by normal brain MRS or by normal molecular genetic investigations (Fig. 1 and Table 3). We depicted low urine guanidinoacetate levels for three patients who had normal brain MRS and molecular genetic investigations in Additional file 3.

Elevated urine creatine was identified in 24% (72/297) of the patients and CRTR deficiency was excluded in 53% (38/72) of males and females either by normal brain MRS or by normal molecular genetic investigations or both (Fig. 1 and Table 4). We depicted the number of elevated urine creatine and the range of elevations in patients who had normal brain MRS, and/or normal molecular genetic investigations in Additional file 4. Two patients with CRTR deficiency diagnosed during the study period were summarized above.

**Table 4:** Elevated urine creatine levels and their follow-up investigations

Urine creatine reference range per age groups	Number of elevated urine creatine levels in males and females	Elevated urine creatine range (mmol/mol creatinine) (median)
		Follow-up investigations
0–60 months (reference range 14–830 mmol/mol creatinine)	Male	838.0–3915.00 (1082.5)
	N = 29	Normal MRS n = 8
	No confirmatory follow-up investigations n = 9	Normal genetic investigations for CRTR deficiency n = 12
	Female	843.0–2333.0 (1190.0)
	N = 25	Normal MRS n = 6
	No confirmatory follow-up investigations n = 13	Normal genetic investigations for CRTR deficiency n = 12
60–192 months (reference range 16–649 mmol/mol creatinine)	Male	707.0–3518.0* (835)
	N = 7	Normal MRS n = 1
	No confirmatory follow-up investigations n = 3	Normal genetic investigations for CRTR deficiency n = 2
		Diagnosis of CRTR deficiency n = 1
	Female	657.0–8756.0** (885)
	N = 9	Normal MRS n = 1
	No confirmatory follow-up investigations n = 6	Normal genetic investigations for CRTR deficiency n = 2
		Diagnosis of CRTR deficiency n = 1
192–228 months (reference range 19–173 mmol/mol creatinine)	Male	531
	N = 1	Normal genetic investigations for CRTR deficiency n = 1
	Female	Result = 186.0
	N = 1	
	No confirmatory follow-up investigations n = 1	

*Maximum value is male patient identified with CRTR without therapy

**Maximum value is female patient identified with CRTR with therapy

In the remaining 295 patients with no CCDD, all patients had neurodevelopmental disorders. Seizures were present in 42% of the patients. Behavioral disorders reported in 26% of the patients. Movement disorders were present in 17% of the patients and ataxia was the most common movement disorder.

We summarized the clinical features, neuroimaging and molecular genetic investigations of 58 patients with genetic diagnoses and CRTR deficiency in Additional file 1. Thirty-seven of these patients underwent a urine creatine metabolite panel for diagnostic investigations of neurodevelopmental disorders and 21 of these patients had a urine creatine metabolite panel for monitoring of creatine metabolism due to their known inherited metabolic disorder. We depicted 35 different genetic diagnoses (except CRTR deficiency) and the number of patients for each in Fig. 2. The most common genetic disease was ornithine aminotransferase deficiency in our study cohort. During the monitoring of creatine metabolism in known inherited metabolic disorders, we found abnormal urine guanidinoacetate and/or urine creatine in several patients. Results of all of these patients are listed in Additional file 1.

## Discussion

In our retrospective cohort study, we report a diagnostic yield of 0.67% (2/297) for the urine creatine metabolite panel if it would have been used as a first-line screening tool during the study period of 6 years. Two patients that identified with CRTR deficiency during the study period had non-targeted molecular genetic investigations: one next generation sequencing panel for intellectual disability and one WES as a first-line investigation, which led to the biochemical confirmation of CRTR deficiency. Interestingly, the female patient had a markedly elevated urine creatine. There were 6 additional patients with CRTR deficiency who underwent the urine creatine metabolite panel for monitoring. Altogether, the prevalence of creatine transporter deficiency was 2.64% (8/303) in our study cohort of patients with neurodevelopmental disorders who underwent screening or monitoring of CCDS at our institution.

In our study, the diagnostic yield of WES was 27% (14/52) in patients with neurodevelopmental disorders [CRTR deficiency (n = 1); *ARX* disease (n = 1); *KDM5C* disease (n = 1); *GABRB3 *disease (n = 1); *KIAA2022 *disease (n = 1); *KCNA2 *disease (n = 1); *PMM2*-CDG (n = 2); *SYNGAP1 *disease (n = 1); Angelman syndrome (n = 1); *PURA *disease (n = 1); *EARS *disease (n = 1); *HECW2 *disease (n = 1); *KAT6B *disease (n = 1)]. Interestingly, the diagnostic yield of WES for CRTR deficiency was 1.9% (1/52) in our study. The diagnostic yield of WES seems to be higher than the urine creatine metabolite panel in the diagnosis of CRTR deficiency (2/52 vs 2/297).

Previously, we reported a 1–30-fold elevation of urine guanidinoacetate levels in molecular genetically confirmed patients with GAMT deficiency [7]. In our current study, urine guanidinoacetate levels were elevated up to 4.4-fold above the upper reference range in 25 patients. We excluded GAMT deficiency based on the normal brain MRS and/or molecular genetic investigations in 52% of those patients. We did not have any true positive GAMT deficiencies in our study cohort, and we are not certain if elevated urine guanidinoacetate had good sensitivity. GAMT deficiency is unlikely to be missed by urine creatine metabolite panel, but a positive test needs to be investigated further. We have two true positive CRTR deficiencies in our study cohort; markedly elevated urine creatine is likely highly sensitive. CRTR deficiency is not missed by urine creatine metabolite panel, but a positive test needs to be investigated further.

Secondary cerebral creatine deficiency was previously reported in argininosuccinate lyase, argininosuccinate synthetase, ornithine aminotransferase and Δ[1]-pyrroline-5-carboxylate synthetase deficiencies [8–10]. Urine guanidinoacetate measurements were helpful to identify some of the secondary creatine deficiency disorders including ornithine-δ-aminotransferase deficiency and ornithine transcarbamylase deficiency in our study. In ornithine-δ-aminotransferase deficiency, low creatine levels are due to two mechanisms: (1) elevated plasma ornithine inhibits AGAT enzyme and decreases guanidinoacetate synthesis; (2) high ornithine competes with arginine to decrease substrate supply to creatine synthesis. In proximal urea cycle disorders, arginine deficiency results in a lack of arginine supply to creatine biosynthesis and decreased guanidinoacetate and creatine synthesis. Low urine guanidinoacetate levels will likely suggest not only AGAT deficiency, but also ornithine-δ-aminotransferase deficiency, proximal urea cycle disorders, as well as failure to thrive and decreased protein intake [11].

In our recent study, males with molecular genetically confirmed CRTR deficiency had 1–12-fold elevation of urine creatine [6]. In our current study, some of the patients had up to a fivefold elevation of urine creatine with normal brain MRS or normal molecular genetic investigations excluding the CRTR deficiency. It seems that there is some overlap between true CRTR deficiency and false positive elevation of creatine.

It should be noted that our study had several limitations including: (1) It is a retrospective cohort study consisting of a chart review of patients that underwent a urine creatine metabolite panel for screening of CCDD at our institution; (2) Despite abnormal urine creatine metabolite panel results being identified in 103 patients, there were no brain MRS or molecular genetic investigations in 49 of those patients. We are not certain if those patients with an abnormal urine creatine metabolite panel might have had CCDD; (3) Due to the lack of confirmatory investigations in 49 patients, we were not able to estimate the true prevalence or the false positive rate of CCDD using the urine creatine metabolite panel or update our reference range to decrease the false positive rate for CCDD; (4) Due to lack of confirmatory investigations in 49 patients, we were not able to calculate the sensitivity and specificity of the urine creatine metabolite panel; (5) In our recent study, WES was applied to 109 children with epilepsy and none of the patients had CCDD [12]. Whereas in our current study, the diagnostic yield of WES was 1.9% for CRTR deficiency, which is unlikely to be a true prevalence of CRTR deficiency in patients with neurodevelopmental disorders who underwent WES; 6) Two of the study authors (S.M-A and A.S.) receive referrals for all patients with CCDD for their management. In more than a 10 years’ time period, there were only 8 patients (including the two patients identified during this study period) known to the authors at our institution. To the best of our knowledge, those are the only patients identified either by a urine creatine metabolite panel, by brain MRS or by molecular genetic investigations over the period of more than 10 years.

In conclusion, we report a low diagnostic yield of the urine creatine panel in patients with neurodevelopmental disorders. Although the diagnostic yield of the urine creatine metabolite panel as a screening tool for neurodevelopmental disorders is low, the low cost and non-invasive nature make urine testing a suitable first-tier test in neurodevelopmental disorders. To the best of our knowledge, this study is the first Canadian study to report on the diagnostic yield of the urine creatine panel for CCDD from a single center. Despite several limitations, our study results are valuable in evaluating the clinical use of a urine creatine panel to identify treatable CCDD.

## Supplementary information

**Additional file 1.** Demographics, clinical features, urine creatine panel, neuroimaging and molecular genetic test results of patients who have undergone urine creatine panel and diagnosed with other genetic diagnoses are listed in Supplemental Table 1.

**Additional file 2.** Elevated urine guanidinoacetate levels in patients, who had normal brain MRS or molecular genetic investigations or both investigations, are depicted in Supplemental Figure 1. Red line shows upper limit of reference range.

**Additional file 3.** Low urine guanidinoacetate levels in patients, who had normal brain MRS or molecular genetic investigations or both investigations, are depicted in Supplemental Figure 2. Red line shows lowest limit of reference range.

**Additional file 4.** Elevated urine creatine in patients, who had normal brain MRS or molecular genetic investigations or both investigations, are depicted in Supplemental Figure 3. Red line shows upper limit of reference range.

## Data Availability

The authors confirm that the part of the data supporting the findings of this study are available within the article [and/or] its supplementary material based on the Research Ethics Board approval. The data that support the findings of this study analysis are available on request from the corresponding author. The data are not publicly available due to the data containing research participants’ information which could compromise the privacy of the participants.

## References

[1] Mercimek-Mahmutoglu S, Salomons GS. Creatine Deficiency Syndromes. In: Adam MP, Ardinger HH, Pagon RA, Wallace SE, Bean LJH, Stephens K, et al., editors. GeneReviews(®). Seattle (WA): University of Washington, Seattle Copyright © 1993–2020, University of Washington, Seattle. GeneReviews is a registered trademark of the University of Washington, Seattle. All rights reserved.; 1993.

[2] El-Gharbawy AH, Goldstein JL, Millington DS, Vaisnins AE, Schlune A, Barshop BA (2013). Elevation of guanidinoacetate in newborn dried blood spots and impact of early treatment in GAMT deficiency. Mol Genet Metab.

[3] Schulze A, Hoffmann GF, Bachert P, Kirsch S, Salomons GS, Verhoeven NM (2006). Presymptomatic treatment of neonatal guanidinoacetate methyltransferase deficiency. Neurology.

[4] Stockler-Ipsiroglu S, Apatean D, Battini R, DeBrosse S, Dessoffy K, Edvardson S (2015). Arginine:glycine amidinotransferase (AGAT) deficiency: Clinical features and long term outcomes in 16 patients diagnosed worldwide. Mol Genet Metab.

[5] Carling RS, Hogg SL, Wood TC, Calvin J (2008). Simultaneous determination of guanidinoacetate, creatine and creatinine in urine and plasma by un-derivatized liquid chromatography-tandem mass spectrometry. Ann Clin Biochem.

[6] Bruun TUJ, Sidky S, Bandeira AO, Debray FG, Ficicioglu C, Goldstein J (2018). Treatment outcome of creatine transporter deficiency: international retrospective cohort study. Metab Brain Dis.

[7] Khaikin Y, Sidky S, Abdenur J, Anastasi A, Ballhausen D, Buoni S (2018). Treatment outcome of twenty-two patients with guanidinoacetate methyltransferase deficiency: An international retrospective cohort study. Eur J Paediat Neurol.

[8] van Spronsen FJ, Reijngoud DJ, Verhoeven NM, Soorani-Lunsing RJ, Jakobs C, Sijens PE (2006). High cerebral guanidinoacetate and variable creatine concentrations in argininosuccinate synthetase and lyase deficiency: implications for treatment?. Mol Genet Metab.

[9] Martinelli D, Häberle J, Rubio V, Giunta C, Hausser I, Carrozzo R (2012). Understanding pyrroline-5-carboxylate synthetase deficiency: clinical, molecular, functional, and expression studies, structure-based analysis, and novel therapy with arginine. J Inherit Metab Dis.

[10] Nänto-Salonen K, Komu M, Lundbom N, Heinänen K, Alanen A, Sipilä I (1999). Reduced brain creatine in gyrate atrophy of the choroid and retina with hyperornithinemia. Neurology.

[11] Brosnan ME, Brosnan JT (2016). The role of dietary creatine. Amino Acids.

[12] Costain G, Cordeiro D, Matviychuk D, Mercimek-Andrews S (2019). Clinical application of targeted next-generation sequencing panels and whole exome sequencing in childhood epilepsy. Neuroscience.

