# Supplementary material for: Urine creatine metabolite panel as a screening test in neurodevelopmental disorders
Source: Orphanet J Rare Dis. 2020 Dec 2;15:339. doi: 10.1186/s13023-020-01617-z (PMC7709238; doi:10.1186/s13023-020-01617-z)
Supplement: Supplementary file 1 — Additional file 1. Demographics, clinical features, urine creatine panel, neuroimaging and molecular genetic test results of patients who have undergone urine creatine panel and diagnosed with other genetic diagnoses are listed in Supplemental Table 1. [file 13023_2020_1617_MOESM1_ESM.docx]

**Supplemental Table 1.** Demographics, clinical features, urine creatine panel, neuroimaging and molecular genetic test results of patients who have undergone urine creatine panel and diagnosed with other genetic diagnoses are listed in Supplemental Table 1.

| **Number/study ID/current age/ sex/ genetic diagnosis (reference)** | **Age of onset/age of diagnosis** | **Clinical features** | **Urine creatine panel** | **Brain MRI/ MRS (age)** | **Molecular genetic test results (references)** |
| --- | --- | --- | --- | --- | --- |
| 1/CCD056/10yrs/F/OAT deficiency | 4yrs/8yrs | Visual impairment | ↓GAA (5; reference range 15-30) | N/N | Compound HTZ novel c.1276C>T (p.Arg426*)/ novel c710G>T (p.G237V) in *OAT* |
| 2/CDD122/15yrs/M/ OAT deficiency | 4yrs/14yrs | Myopia, visual impairment | ↓Creatine (8; reference range 16-649)  ↓GAA (8; reference range 15-30) | Optic nerve elongation and tortuosity/N (14yrs) | Compound HTZ known c.897C>G (p.Tyr299)/known c.1058 (p.Gly353Asp) in *OAT* (Mashima et al., 1992) |
| 3/CCD123/13yrs/F/ OAT deficiency | 3yrs/12yrs | Myopia, visual impairment | ↓Creatine (8; reference range 16-649)  ↓GAA (8; reference range 15-30) | N/NP | Compound HTZ known c.897C>G (p.Tyr299*)/known c.1058 (p.Gly353Asp) in *OAT* (Mashima et al., 1992) |
| 4/CCD204/17yrs/F/OAT deficiency | 3yrs/16yrs | GDD, visual impairment | ↓Creatine(1.0; reference range 19-173)  ↓GAA (7.4; reference range 11-60) | Macrophthalmia with flattening of post sclera/ low creatine (14yrs) | HMZ novel c.899C>G (p.Pro300Arg) in *OAT* |
| 5/CCD205/12yrs/M/ OAT deficiency | 3yrs/8yrs | GDD, visual impairment | ↓Creatine (2; reference range 16-649)  ↓GAA (<1; reference range 15-130) | Thin CC (9yrs)/N | HMZ novel c.899C>G (p.Pro300Arg) in *OAT* |
| 6/CCD206/20 yrs/M/ OAT deficiency | 3yrs/16yrs | GDD, visual impairment | ↓Creatine (7; reference range 19-173)  ↓GAA (1; reference range 11-60) | Thin CC, cerebral atrophy (16yrs)/N | HMZ novel c.899C>G (p.Pro300Arg) in *OAT* |
| 7/CCD243/15yrs/M/OAT deficiency | 3yrs/15yrs | Visual impairment | ↓Creatine (4; reference range 16-649)  ↓GAA (9; reference range 15-130) | N/low creatine, elevated NAA (13yrs) | HMZ novel c.899C>G (p.Pro300Arg) in *OAT* |
| 8/CCD039/12yrs/F/*SCN1A* disease  (Costain et al 2019) | 6mo/12yrs | GDD, epilepsy (GTCS, FS, AS), hypotonia,  movement disorder (ataxia, tremor), behavioural disorder (aggression, ASD) | ↓Creatine (8; reference range 16-649) | NP | HTZ novel c.3429G>A (p.Glu1143Glu) in *SCN1A* |
| 9/CCD107/7yrs/F/*SCN1A* disease (Mercimek-Mahmutoglu et al., 2015) | 4 mo/2 yrs | GDD, epilepsy (FS), behavioural disorder | ↑Creatine (2333; reference range 14-830)  ↑GAA (172; reference range 5-150) | N | HTZ novel c.482G>A (p.Gln1815*) in *SCN1A* |
| 10/CCD114/15yrs/F/*SCN1A* disease  (Costain et al 2019) | 7days/12yrs | GDD, epilepsy, microcephaly, ASD | ↑Creatine (883; reference range 16-649)  ↑GAA (132; reference range 15-130) | PVL (11.5yrs)/ NP | HTZ novel c.739_744delCTCTCAinsAGC (p.Leu247del) in *SCN1A* |
| 11/CCD258/4yrs/M/ *SCN1A* disease  (Costain et al 2019) | 5mo/2yrs | GDD, epilepsy (FS, GTCS, hemiclonic) | ↑Creatine(958; reference range 14-830) | N (2yrs) | HTZ novel c.2665G>A (p. Ala889Thr) in *SCN1A* |
| 12/CCD031/13yrs/M/Angelman syndrome | 22mo/13yrs | GDD, epilepsy (AS, CPS) behavioural disorder (aggression, ADHD), movement disorder (tremor) | ↑Creatine (958; reference range 16-649) | N | HTZ novel c.72-12T>A in *UBE3A* |
| 13/CDD199/8yrs/M/Angelman syndrome | Neonatal/7yrs | GDD, epilepsy (AbS), FTT,  dysmorphic features, microcephaly | N | NP | HTZ novel c.8 G>A (p.Arg2Gln) in *UBE3A* |
| 14/CCD279/6yrs/M/Angelman syndrome | 7mo/6yrs | GDD, epilepsy (AS), hypotonia | N | Thin CC (4.5mo)/NP | HTZ novel n c.1396A>G (p.Lys466Glu) in *UBE3A* |
| 15/CCD280/6yrs/M/Angelman syndrome | 7mo/6yrs | GDD, epilepsy, hypotonia | N | Hypoplastic CC (4.5mo)/NP | HTZ novel c.1396A>G (p.Lys466Glu) in *UBE3A* |
| 16/CCD045/13yrs/F/citrullinemia type I | 17mo/4yrs | GDD, liver failure | N | N (3 yrs)/NP | Compound HTZ novel IVS4-119G>A (g.4967G/ known c.1087xC>T (p.Arg363Trp) in *ASS1* (Haberle et al., 2002) |
| 17/CCD181/4yrs/M/citrullinemia type I | 2days/1mo | GDD, epilepsy, hypotonia | N | Delayed myelination, cerebral atrophy (8mo)/NP | HMZ known c.470G>A (p.R157H) in *ASS1* (Kobayashi et al., 1990) |
| 18/CCD229/3yrs/M/ citrullinemia type I | Neonatal/1yr | FTT | ↑Creatine(958; reference range 14-30) | NP | Compound HTZ known c.787G>A (p.Val263Met)/ known c.971G>T (p.Gly324Val) in *ASS1*  (Häberle et al., 2003) |
| 19/CCD289/11 yrs/F/ *WDR62* disease | Neonatal/11.5yrs | GDD, microcephaly, dysmorphic features | N | NP | HMZ novel c.1589dupT (p.Glu531Glyfs*4) in *WDR62* |
| 20/CCD290/11 yrs/F/ *WDR62* disease | 1 week/4.5 yrs | GDD, hypotonia, microcephaly, hearing loss, behaviour disorder, dysmorphic features | N | Diffuse cortical thickening (5 yrs)/NP | HMZ novel c.1589dupT (p.Glu531Glyfs*4) in *WDR62* |
| 21/CCD149/5yrs/M/Prader-Willi syndrome | Neonatal/2mo | Hypotonia, dysmorphic features | ↑GAA (281; reference range 5-150) | N (newborn)/NP | Hypermethylation of *SNRPN* |
| 22/CCD301/22mo/M/Prader-Willi syndrome | Neonatal/2 mo | GDD, hypotonia, dysmorphic features, FTT | N | N | Hypermethylation of *SNRPN* |
| 23/CCD129/6yrs/M/*PMM2-*CDG | 11mo/5yrs | GDD, hypotonia, behavioural disorder (aggression), movement disorder (ataxia) | N | Cerebellar hypoplasia (4.5yrs)/NP | Compound HTZ known pathogenic c.422G>A (p.Arg141His)/c.323C>T (p.Ala108Val) in *PMM2* (Matthijis et al., 1997) |
| 24/CCD215/4yrs/F/*PMM2-*CDG | Neonatal/3yrs | GDD, epilepsy, hypotonia, movement disorder (ataxia) | ↑Creatine(906; reference range 14-830) | Cerebellar atrophy (18mo)/NP | Compound HTZ known c.422G>A (p.Arg141His)/ known c.323C>T (p.Ala108Val) in *PMM2* (Matthijs et al., 1997) |
| 25/CCD010/19yrs/M/ *TUBB2B* disease (Mercimek-Mahmutoglu, 2015) | 3yrs/12yrs | GDD, ASD | N | Parahippocampal polymicrogyria (2 yrs)/ NP | 1 Mb deletion in 6p25.2 involving *TUBB2B* |
| 26/CCD160/7yrs/M/*SYNGAP1* disease | 9mo/5yrs | GDD, epilepsy (MS), dysmorphic features, hypotonia, movement disorder (ataxia) | N | N | HTZ novel c.1393delC (p.Lys465PhefsX9) in *SYNGAP1* |
| 27/CCD267/3 yrs/M/ *STXBP1* disease  (Costain et al 2019) | 7week/3yrs | GDD, epilepsy (AbS, GTCS, GTS) | ↓Creatine (4; reference range 14-830) | N | HTZ known c.874C>T (p.Arg292Cys) in *STXBP1* (Stamberger et al., 2016) |
| 28/CCD161/7 yrs/F/  *SLC13A5* disease  (Costain et al 2019) | Neonatal/5.5yrs | GDD, epilepsy, FTT, microcephaly, movement disorder (dystonia, ataxia, choreoathetosis) | N | N | HMZ novel c.716+5G>A in *SLC13A5* |
| 29/CCD269/3yrs/F/ *PYCR2* disease  (Costain et al., 2019) | 8mo/2yrs | GDD, epilepsy (MS), FTT, hypotonia, dysmorphic features | N | Delayed myelination, thin CC (8mo)/NP | HMZ known c.796C>T (p.Arg266*) in *PYCR2* |
| 30/CCD260/3yrs/F/*PURA* disease | 18 days/3yrs | GDD, FTT, hypotonia | ↑Creatine(1382; reference range 14-830) | NP | HTZ novel c.1594dupG (p.Leu54AlafsX147) in *PURA* |
| 31/CCD001/24yrs/F/OTC deficiency | Newborn/birth | None | ↓Creatine (4; reference range 14-830)  ↓GAA (14; reference range 5-150) | NP | Hemizygous known c.95T>C (p.Leu95Ser) in *OTC* (Mew et al., 2017) |
| 32/CCD175/6 yrs/M/  MTHFR deficiency | 4mo/1.5yrs | GDD, epilepsy (AbS),  hypotonia, ASD | N | Delayed myelination, cerebral atrophy (3yrs)/NP | Compound HTZ known c.1408G>T (p.Glu470*) (Homberger et al., 2000)/ known c.1760A>T (p.Lys584*) in *MTHFR* (Sibani et al., 2000) |
| 33/CCD011/19yrs/M/MELAS | 8yrs/10 yrs | GDD, epilepsy (CPS), hemiparesis | ↑Creatine(4481; reference range 16-649) (on creatine supplementation) | Brain edema and increased signal in left parietal in FLAIR/increased lactate in left basal ganglia (11.5 yrs) | Known m.3243A>G in mt*TL1* (El-Hattab et al., 2015) |
| 34/CCD038/12yrs/F/MAT I/III def | Newborn/11 yrs | Normal | ↓Creatine (12; reference range 16-649) | Increased T2 signal in cerebral frontal and parietal WM (10.5yrs) | HMZ novel c.A95C>D (p.R299C) in *MAT1A* |
| 35/CCD239/3yrs/M/Marfan syndrome | Neonatal/3mo | GDD, FTT, hypotonia, dysmorphic features, microcephaly | N | Decreased WM, thin CC (16mo)/NP | 6.907 Mb known deletion in 15q15.1q21.1 involving *FBN1* (Hilhorst-Hofstee et al., 2011) |
| 36/CCD306/2 yrs/M/ Krabbe disease | 7.5 mo/1 yr | GDD, epilepsy (GTCS, AbS, AS) | ↓Creatine (13; reference range 14-830) | Symmetrical increased signal in WM in T2/NP | HMZ known c.1162_2058del11897 in *GALC* |
| 37/CCD110/18yrs/F/  *KIAA2022* disease | 11yrs/17.5 yrs | GDD, epilepsy | ↓Creatine (5; reference range 19-173)  ↓GAA (11; reference range 11-60) | Increased signal in left hippocampus in FLAIR and T2 (13.5yrs)/NP | HTZ novel c.336G>A (p.Trp112*) in *KlAA2022* |
| 38/CCD068/12yrs/F/*KDM5C* disease | Neonatal/12yrs | GDD, ID, FTT,  behavioral disorder (ADHD) | ↑Creatine(885; reference range 16-649) | N | HTZ novel c.1613C>T (p.Pro538Leu) in *KDM5C* |
| 39/CCD111/14yrs/F/*KCNA2* disease  (Costain et al, 2019) | 8mo/10.5yrs | GDD, epilepsy (AbS, GTCS), hypotonia, movement disorder (ataxia, tremor) | N | Cerebellar atrophy (10yrs)/ NP | HTZ known c.890G>A (p.Arg297Gln) in *KCNA2* (Pena et al., 2015) |
| 40/CCD299/2 yrs/M/ *KAT6B* disease | 10mo/15mo | GDD, hypotonia, ptosis, dysmorphic features | ↑GAA (188; reference range 5-150) | NP | HTZ known c.4205_4206delCT (p.Ser1402CysfsX5) in *KAT6B* (Clayton-Smith et al., 2011) |
| 41/CCD286/2 yrs/M/ *HECW2* disease | 6mo/2yrs | GDD, optic atrophy, hypotonia | ↑GAA (133; reference range 5-150) | N (6mo)/NP | Novel c.3572G>A (p.Arg1191Gln) in *HECW2* |
| 42/CCD072/10yrs/F/*GABRB3* disease  (Costain et al, 2019) | 1yr/8yrs | GDD, epilepsy (Abs, GTCS), movement disorder (tremor) | N | N | HTZ novel c485T>C (p.Met162Thr) in *GABRB3* |
| 43/CCD277/6 yrs/F/ *EARS2* disease  (Costain et al., 2019) | Neonatal/4yrs | GDD, epilepsy, hypotonia, movement disorder (dystonia) | N | Increased signal in globus pallidus bilaterally in T2/FLAIR (4yrs)/NP | HMZ novel c.293C>T (p.Ala98Val) in *EARS2* |
| 44/CCD312/2 yrs/F/ *DARS*  disease  (Taft et al., 2013) | 6mo/14mo | GDD, hypotonia, FTT | ↑Creatine (1247; reference range 14-830) | Increased WM signal/N (56 weeks) | HMZ known c.766A>C (p.Met255Leu) in *DARS* |
| 45/CCD154/4yrs (deceased)/F/Cockayne syndrome | Neonatal/ 3yrs | GDD, FTT, microcephaly, dysmorphic features | N | Delayed myelination (1.5yrs)/NP | Compound HTZ known c.2287-2A>G (Laugel et al., 2009)/ novel c.1442dupA in *ERCC6* |
| 46/CCD002/21yrs/M/ Bartter syndrome | 2.5yrs/ 13.5yrs | Polyuria, polydipsia, hypokalemia, nephrocalcinosis | ↓Creatine (12; reference range 16-649)  ↓GAA (13; reference range 15-130) | PVL, thin CC (14 yrs)/ NP | HMZ known c.1432G>A (p.Gly478Arg) in *SLC12A1* (Vargas-Poussou et al., 1998) |
| 47/CCD009/19yrs/F/*ATP1A3* disease  (Costain et al., 2019) | 1.5mo/15yrs | GDD, epilepsy (AbS, GTCS, febrile), FTT, behavioral disorder (aggressive) paroxysmal hemiparesis, hypotonia, movement disorder (ataxia, dystonia) | ↓Creatine (13; reference range 16-649) | Cerebral atrophy (9yrs)/ NP | HTZ novel c.2840 G>A (p.Gly947Glu) in *ATP1A3* |
| 48/CCD097/10yrs/M/Ataxia-Telangiectasia (Cordeiro et al., 2018) | 2 yrs/4 yrs 9 mo | GDD, microcephaly, hypotonia, movement disorder (ataxia) | N | NP | HMZ known c.590G>A (p.Gly197Glu) in *ATM* |
| 49/CCD026/16yrs/M/  *ARX* disease  (Breen et al., 2018) | 6mo/14yrs | GDD, hypotonia, behavioral disorder (aggression) movement disorder (dystonia) | N | Focal cortical dysplasia of the right frontal lobe (8.5 yrs)/NP | Hemizygous known c.426_458dup (p.Gly143_Ala153dup) in *ARX* |
| 50/CCD187/15yrs/M/arginase I deficiency | 5mo/NA | GDD, ID, epilepsy (MS, GTCS, AbS, AS), hypotonia | ↑Creatine(1696; reference range 16-649)  ↑GAA (288; reference range 15-130) | Increased signal in WM in FLAIR, cerebellum atrophy(11.5yrs)/low NAA | Compound HTZ known (Uchino et al., 1992) c.365G>A (p.Trp122Ter)/ novel c.23T>A (p.Ile8Lys) in *ARG1* |
| 51/CCD088/8yrs/F/*POLG* disease | 1yr/5yrs | Cognitive dysfunction, epilepsy (GTCS) | ↑GAA (135; reference range 15-130) | Increased signal in right temporal WM (5.5 yrs)/NP | Compound HTZ known c.2209G>C (p.Gly737Arg)/ c.3139C>T (p.Arg1047Trp) in *POLG* (Wiltshire et al., 2008) |
| 52/CCD057/11yrs/M/ 2p16.3 deletion syndrome | Neonatal/6yrs | GDD, hypotonia, movement disorder (ataxia) | N | N | 0.337 MB deletion in 2p16.3 involving *NRXN1* (Bena et al., 2013) |
| 53//CCD006/22yrs/M/ CRTR deficiency (Bruun et al 2018) | 2 yrs/11 yrs 9 mo | GDD, ID, behavioral disorder (aggressive) | ↑Creatine (3552; reference range 14-830)  N guanidinoacetate (83 mmol/mol creatinine; reference range 5-150) | N/absent creatine peak (6yrs) | Hemizygous novel IVS9+24del 24bp in *SCL6A8* |
| 54/CCD058/10 yrs/M/ CRTR deficiency (Bruun et al 2018) | 13 mo/2 yrs | GDD, epilepsy (GTCS), hypotonia, behavioral disorder (ASD, ADHD), movement disorder (ataxia) | ↑Creatine (4834; reference range 14-830)  N guanidinoacetate (36 mmol/mol creatinine; reference range 5-150) | PVL, thin CC/absent creatine peak (18 mo) | Hemizygous novel c.1684dupT (p.Trp562Lysfs*28) in *SLC6A8* |
| 55/ CCD070/11 yrs/M/CRTR deficiency | 14 mo/2 yrs | GDD, epilepsy (FS, GTCS) | ↑Creatine (9826; reference range 14-830)  (on creatine supplementation)  N guanidinoacetate (71 mmol/mol creatinine; reference range 5-150) | NP | Hemizygous novel c.917G>A (p.Trp306*) in *SLC6A8* |
| 56/CCD086/9 yrs/M/ CRTR deficiency (Bruun et al 2018) | Neonatal/2.5 yrs | GDD, ID, hypotonia, behavioral disorder (ASD, aggression, HA), movement disorder (ataxia) | ↑Creatine (3029; reference range 14-830)  N guanidinoacetate (34 mmol/mol creatinine; reference range 5-150) | PVL/Absent creatine peak (21 mo) | Hemizygous c.634G>T (p.Glu212*) in *SLC6A8* |
| 57//CCD093/19 yrs/F/ CRTR deficiency (Bruun et al., 2018) | 2.5 yrs/6yrs | GDD, ID, epilepsy (GTCS), behavioral disorder (ADHD) | ↑Creatine(1721; reference range 16-649)  N guanidinoacetate (127 mmol/mol creatinine; reference range 5-130) | Increased T2 signal in subcortical, periventricular WM/decreased creatine peak | Heterozygous c.1067G>T (p.Gly356Val) in *SLC6A8* |
| 58/CCD094/17 yrs/F/CRTR deficiency  (Bruun et al., 2018) | 5 yrs/8 yrs | GDD, ID, epilepsy (GTC, AS), behavioral disorder (ADHD) | ↑Creatine (1865; reference range 16-649)  N guanidinoacetate (106 mmol/mol creatinine; reference range 5-130) | Increased signal in white matter/partially absent creatine (6 yrs) | Heterozygous known c.1067G>T (p.Gly356Val) in *SLC6A8* |

**Abbreviations (listed alphabetically):** AbS=absence seizures; ADHD=attention deficient hyperactivity disorder; AS=atonic seizures; ASD=autism spectrum disorder; CC=corpus callosum; CDG=congenital disorders of glycosylation; CPS=complex partial seizures; def=deficiency; FS=febrile seizures; FTT=failure to thrive; GAA=guanidinoacetic acid GDD=global developmental delay; GTCS=generalized tonic-clonic seizures; GTS=generalized tonic seizures; G6PD=glucose-6-phosphate dehydrogenase; mo=month(s); HMZ=homozygous; HTZ=heterozygous; ID=intellectual disability; MELAS=mitochondrial myopathy, encephalopathy, lactic acidosis, and stroke; Met=methionine; MRI=magnetic resonance imaging; MRS=magnetic resonance spectroscopy; MS=myoclonic seizures; N=normal; NA=not available; NP=not performed; OAT=ornithine aminotransferase; PVL=periventricular leukomalacia; WM=white matter; yrs=year(s)

**References**

([1](#_ENREF_1))

([2](#_ENREF_2))

([3](#_ENREF_3))

([4](#_ENREF_4))

([5](#_ENREF_5))

([6](#_ENREF_6))

([7-12](#_ENREF_7))

([13-26](#_ENREF_13))

([27-44](#_ENREF_27))

([45-64](#_ENREF_45))

**References**

1. Aretz S, Stienen D, Uhlhaas S, Loff S, Back W, Pagenstecher C, et al. High proportion of large genomic STK11 deletions in Peutz-Jeghers syndrome. Human mutation. 2005;26(6):513-9.

2. Beggs AD, Latchford AR, Vasen HF, Moslein G, Alonso A, Aretz S, et al. Peutz-Jeghers syndrome: a systematic review and recommendations for management. Gut. 2010;59(7):975-86.

3. Béna F, Bruno DL, Eriksson M, van Ravenswaaij-Arts C, Stark Z, Dijkhuizen T, et al. Molecular and clinical characterization of 25 individuals with exonic deletions of NRXN1 and comprehensive review of the literature. American journal of medical genetics Part B, Neuropsychiatric genetics : the official publication of the International Society of Psychiatric Genetics. 2013;162b(4):388-403.

4. Bhat YR, Vinayaka G, Sreelakshmi K. Antenatal bartter syndrome: a review. International journal of pediatrics. 2012;2012:857136.

5. Bonomi M, Rochira V, Pasquali D, Balercia G, Jannini EA, Ferlin A. Klinefelter syndrome (KS): genetics, clinical phenotype and hypogonadism. Journal of endocrinological investigation. 2017;40(2):123-34.

6. Brashear A, Sweadner KJ, Cook JF, Swoboda KJ, Ozelius L. ATP1A3-Related Neurologic Disorders. In: Adam MP, Ardinger HH, Pagon RA, Wallace SE, Bean LJH, Stephens K, et al., editors. GeneReviews(®). Seattle (WA): University of Washington, Seattle

Copyright © 1993-2020, University of Washington, Seattle. GeneReviews is a registered trademark of the University of Washington, Seattle. All rights reserved.; 1993.

7. Breen DP, Mercimek-Andrews S, Lang AE. Infantile-onset hand dystonia with intellectual disability: Clues to ARX mutations. Neurology. 2018;90(7):333-5.

8. Lemire G, Campeau PM, Lee BH. KAT6B Disorders. In: Adam MP, Ardinger HH, Pagon RA, Wallace SE, Bean LJH, Stephens K, et al., editors. GeneReviews(®). Seattle (WA): University of Washington, Seattle

Copyright © 1993-2020, University of Washington, Seattle. GeneReviews is a registered trademark of the University of Washington, Seattle. All rights reserved.; 1993.

9. Bruun TUJ, Sidky S, Bandeira AO, Debray FG, Ficicioglu C, Goldstein J, et al. Treatment outcome of creatine transporter deficiency: international retrospective cohort study. Metabolic brain disease. 2018;33(3):875-84.

10. Carrillo F, Schneider SA, Taylor AM, Srinivasan V, Kapoor R, Bhatia KP. Prominent oromandibular dystonia and pharyngeal telangiectasia in atypical ataxia telangiectasia. Cerebellum (London, England). 2009;8(1):22-7.

11. Catarzi S, Caciotti A, Thusberg J, Tonin R, Malvagia S, la Marca G, et al. Medium-chain acyl-CoA deficiency: outlines from newborn screening, in silico predictions, and molecular studies. TheScientificWorldJournal. 2013;2013:625824.

12. Chen S, Zhang L, Bryant RM, Vincent GM, Flippin M, Lee JC, et al. KCNQ1 mutations in patients with a family history of lethal cardiac arrhythmias and sudden death. Clinical genetics. 2003;63(4):273-82.

13. Chien YH, Abdenur JE, Baronio F, Bannick AA, Corrales F, Couce M, et al. Mudd's disease (MAT I/III deficiency): a survey of data for MAT1A homozygotes and compound heterozygotes. Orphanet journal of rare diseases. 2015;10:99.

14. Clayton-Smith J, O'Sullivan J, Daly S, Bhaskar S, Day R, Anderson B, et al. Whole-exome-sequencing identifies mutations in histone acetyltransferase gene KAT6B in individuals with the Say-Barber-Biesecker variant of Ohdo syndrome. American journal of human genetics. 2011;89(5):675-81.

15. Cordeiro D, Bullivant G, Siriwardena K, Evans A, Kobayashi J, Cohn RD, et al. Genetic landscape of pediatric movement disorders and management implications. Neurology Genetics. 2018;4(5):e265.

16. Costain G, Cordeiro D, Matviychuk D, Mercimek-Andrews S. Clinical Application of Targeted Next-Generation Sequencing Panels and Whole Exome Sequencing in Childhood Epilepsy. Neuroscience. 2019;418:291-310.

17. Dagli AI, Mueller J, Williams CA. Angelman Syndrome. In: Adam MP, Ardinger HH, Pagon RA, Wallace SE, Bean LJH, Stephens K, et al., editors. GeneReviews(®). Seattle (WA): University of Washington, Seattle

Copyright © 1993-2020, University of Washington, Seattle. GeneReviews is a registered trademark of the University of Washington, Seattle. All rights reserved.; 1993.

18. Dean L. Methylenetetrahydrofolate Reductase Deficiency. In: Pratt VM, McLeod HL, Rubinstein WS, Scott SA, Dean LC, Kattman BL, et al., editors. Medical Genetics Summaries. Bethesda (MD): National Center for Biotechnology Information (US); 2012.

19. Demos MK, Fullston T, Partington MW, Gécz J, Gibson WT. Clinical study of two brothers with a novel 33 bp duplication in the ARX gene. American journal of medical genetics Part A. 2009;149a(7):1482-6.

20. Dietz H. Marfan Syndrome. In: Adam MP, Ardinger HH, Pagon RA, Wallace SE, Bean LJH, Stephens K, et al., editors. GeneReviews(®). Seattle (WA): University of Washington, Seattle

Copyright © 1993-2020, University of Washington, Seattle. GeneReviews is a registered trademark of the University of Washington, Seattle. All rights reserved.; 1993.

21. Driscoll DJ, Miller JL, Schwartz S, Cassidy SB. Prader-Willi Syndrome. In: Adam MP, Ardinger HH, Pagon RA, Wallace SE, Bean LJH, Stephens K, et al., editors. GeneReviews(®). Seattle (WA): University of Washington, Seattle

Copyright © 1993-2020, University of Washington, Seattle. GeneReviews is a registered trademark of the University of Washington, Seattle. All rights reserved.; 1993.

22. El-Hattab AW. Systemic Primary Carnitine Deficiency. In: Adam MP, Ardinger HH, Pagon RA, Wallace SE, Bean LJH, Stephens K, et al., editors. GeneReviews(®). Seattle (WA): University of Washington, Seattle

Copyright © 1993-2020, University of Washington, Seattle. GeneReviews is a registered trademark of the University of Washington, Seattle. All rights reserved.; 1993.

23. El-Hattab AW, Almannai M, Scaglia F. MELAS. In: Adam MP, Ardinger HH, Pagon RA, Wallace SE, Bean LJH, Stephens K, et al., editors. GeneReviews(®). Seattle (WA): University of Washington, Seattle

Copyright © 1993-2020, University of Washington, Seattle. GeneReviews is a registered trademark of the University of Washington, Seattle. All rights reserved.; 1993.

24. Häberle J, Pauli S, Linnebank M, Kleijer WJ, Bakker HD, Wanders RJ, et al. Structure of the human argininosuccinate synthetase gene and an improved system for molecular diagnostics in patients with classical and mild citrullinemia. Human genetics. 2002;110(4):327-33.

25. Häberle J, Pauli S, Schmidt E, Schulze-Eilfing B, Berning C, Koch HG. Mild citrullinemia in Caucasians is an allelic variant of argininosuccinate synthetase deficiency (citrullinemia type 1). Molecular genetics and metabolism. 2003;80(3):302-6.

26. Hilhorst-Hofstee Y, Hamel BC, Verheij JB, Rijlaarsdam ME, Mancini GM, Cobben JM, et al. The clinical spectrum of complete FBN1 allele deletions. European journal of human genetics : EJHG. 2011;19(3):247-52.

27. Holder JL, Jr., Hamdan FF, Michaud JL. SYNGAP1-Related Intellectual Disability. In: Adam MP, Ardinger HH, Pagon RA, Wallace SE, Bean LJH, Stephens K, et al., editors. GeneReviews(®). Seattle (WA): University of Washington, Seattle

Copyright © 1993-2020, University of Washington, Seattle. GeneReviews is a registered trademark of the University of Washington, Seattle. All rights reserved.; 1993.

28. Homberger A, Linnebank M, Winter C, Willenbring H, Marquardt T, Harms E, et al. Genomic structure and transcript variants of the human methylenetetrahydrofolate reductase gene. European journal of human genetics : EJHG. 2000;8(9):725-9.

29. Howell KB, McMahon JM, Carvill GL, Tambunan D, Mackay MT, Rodriguez-Casero V, et al. SCN2A encephalopathy: A major cause of epilepsy of infancy with migrating focal seizures. Neurology. 2015;85(11):958-66.

30. Khaikin Y, Mercimek-Mahmutoglu S. STXBP1 Encephalopathy with Epilepsy. In: Adam MP, Ardinger HH, Pagon RA, Wallace SE, Bean LJH, Stephens K, et al., editors. GeneReviews(®). Seattle (WA): University of Washington, Seattle

Copyright © 1993-2020, University of Washington, Seattle. GeneReviews is a registered trademark of the University of Washington, Seattle. All rights reserved.; 1993.

31. Kobayashi K, Jackson MJ, Tick DB, O'Brien WE, Beaudet AL. Heterogeneity of mutations in argininosuccinate synthetase causing human citrullinemia. The Journal of biological chemistry. 1990;265(19):11361-7.

32. Laugel V, Dalloz C, Durand M, Sauvanaud F, Kristensen U, Vincent MC, et al. Mutation update for the CSB/ERCC6 and CSA/ERCC8 genes involved in Cockayne syndrome. Human mutation. 2010;31(2):113-26.

33. Le Bizec C, Vuillaumier-Barrot S, Barnier A, Dupré T, Durand G, Seta N. A new insight into PMM2 mutations in the French population. Human mutation. 2005;25(5):504-5.

34. Lichter-Konecki U, Caldovic L, Morizono H, Simpson K. Ornithine Transcarbamylase Deficiency. In: Adam MP, Ardinger HH, Pagon RA, Wallace SE, Bean LJH, Stephens K, et al., editors. GeneReviews(®). Seattle (WA): University of Washington, Seattle

Copyright © 1993-2020, University of Washington, Seattle. GeneReviews is a registered trademark of the University of Washington, Seattle. All rights reserved.; 1993.

35. Mashima Y, Murakami A, Weleber RG, Kennaway NG, Clarke L, Shiono T, et al. Nonsense-codon mutations of the ornithine aminotransferase gene with decreased levels of mutant mRNA in gyrate atrophy. American journal of human genetics. 1992;51(1):81-91.

36. Merritt JL, 2nd, Chang IJ. Medium-Chain Acyl-Coenzyme A Dehydrogenase Deficiency. In: Adam MP, Ardinger HH, Pagon RA, Wallace SE, Bean LJH, Stephens K, et al., editors. GeneReviews(®). Seattle (WA): University of Washington, Seattle

Copyright © 1993-2020, University of Washington, Seattle. GeneReviews is a registered trademark of the University of Washington, Seattle. All rights reserved.; 1993.

37. Matthijs G, Schollen E, Pardon E, Veiga-Da-Cunha M, Jaeken J, Cassiman JJ, et al. Mutations in PMM2, a phosphomannomutase gene on chromosome 16p13, in carbohydrate-deficient glycoprotein type I syndrome (Jaeken syndrome). Nature genetics. 1997;16(1):88-92.

38. Meng L, Donti T, Xia F, Niu Z, Al Shamsi A, Hertecant J, et al. Homozygous variants in pyrroline-5-carboxylate reductase 2 (PYCR2) in patients with progressive microcephaly and hypomyelinating leukodystrophy. American journal of medical genetics Part A. 2017;173(2):460-70.

39. Mercimek-Mahmutoglu S, Patel J, Cordeiro D, Hewson S, Callen D, Donner EJ, et al. Diagnostic yield of genetic testing in epileptic encephalopathy in childhood. Epilepsia. 2015;56(5):707-16.

40. Ah Mew N, Simpson KL, Gropman AL, Lanpher BC, Chapman KA, Summar ML. Urea Cycle Disorders Overview. In: Adam MP, Ardinger HH, Pagon RA, Wallace SE, Bean LJH, Stephens K, et al., editors. GeneReviews(®). Seattle (WA): University of Washington, Seattle

Copyright © 1993-2020, University of Washington, Seattle. GeneReviews is a registered trademark of the University of Washington, Seattle. All rights reserved.; 1993.

41. Miller IO, Sotero de Menezes MA. SCN1A Seizure Disorders. In: Adam MP, Ardinger HH, Pagon RA, Wallace SE, Bean LJH, Stephens K, et al., editors. GeneReviews(®). Seattle (WA): University of Washington, Seattle

Copyright © 1993-2020, University of Washington, Seattle. GeneReviews is a registered trademark of the University of Washington, Seattle. All rights reserved.; 1993.

42. Orsini JJ, Escolar ML, Wasserstein MP, Caggana M. Krabbe Disease. In: Adam MP, Ardinger HH, Pagon RA, Wallace SE, Bean LJH, Stephens K, et al., editors. GeneReviews(®). Seattle (WA): University of Washington, Seattle

Copyright © 1993-2020, University of Washington, Seattle. GeneReviews is a registered trademark of the University of Washington, Seattle. All rights reserved.; 1993.

43. Papandreou A, McTague A, Trump N, Ambegaonkar G, Ngoh A, Meyer E, et al. GABRB3 mutations: a new and emerging cause of early infantile epileptic encephalopathy. Developmental medicine and child neurology. 2016;58(4):416-20.

44. Pena SD, Coimbra RL. Ataxia and myoclonic epilepsy due to a heterozygous new mutation in KCNA2: proposal for a new channelopathy. Clinical genetics. 2015;87(2):e1-3.

45. Pomponio RJ, Ozand PT, Al Essa M, Wolf B. Novel mutations in children with profound biotinidase deficiency from Saudi Arabia. Journal of inherited metabolic disease. 2000;23(2):185-7.

46. Quinonez SC, Thoene JG. Citrullinemia Type I. In: Adam MP, Ardinger HH, Pagon RA, Wallace SE, Bean LJH, Stephens K, et al., editors. GeneReviews(®). Seattle (WA): University of Washington, Seattle

Copyright © 1993-2020, University of Washington, Seattle. GeneReviews is a registered trademark of the University of Washington, Seattle. All rights reserved.; 1993.

47. Reijnders MRF, Leventer RJ, Lee BH, Baralle D, Selber P, Paciorkowski AR, et al. PURA-Related Neurodevelopmental Disorders. In: Adam MP, Ardinger HH, Pagon RA, Wallace SE, Bean LJH, Stephens K, et al., editors. GeneReviews(®). Seattle (WA): University of Washington, Seattle

Copyright © 1993-2020, University of Washington, Seattle. GeneReviews is a registered trademark of the University of Washington, Seattle. All rights reserved.; 1993.

48. Rothblum-Oviatt C, Wright J, Lefton-Greif MA, McGrath-Morrow SA, Crawford TO, Lederman HM. Ataxia telangiectasia: a review. Orphanet journal of rare diseases. 2016;11(1):159.

49. Saneto RP, Cohen BH, Copeland WC, Naviaux RK. Alpers-Huttenlocher syndrome. Pediatric neurology. 2013;48(3):167-78.

50. Shoubridge C, Fullston T, Gécz J. ARX spectrum disorders: making inroads into the molecular pathology. Human mutation. 2010;31(8):889-900.

51. Sibani S, Christensen B, O'Ferrall E, Saadi I, Hiou-Tim F, Rosenblatt DS, et al. Characterization of six novel mutations in the methylenetetrahydrofolate reductase (MTHFR) gene in patients with homocystinuria. Human mutation. 2000;15(3):280-7.

52. Sparks SE, Krasnewich DM. PMM2-CDG (CDG-Ia). In: Adam MP, Ardinger HH, Pagon RA, Wallace SE, Bean LJH, Stephens K, et al., editors. GeneReviews(®). Seattle (WA): University of Washington, Seattle

Copyright © 1993-2020, University of Washington, Seattle. GeneReviews is a registered trademark of the University of Washington, Seattle. All rights reserved.; 1993.

53. Stamberger H, Nikanorova M, Willemsen MH, Accorsi P, Angriman M, Baier H, et al. STXBP1 encephalopathy: A neurodevelopmental disorder including epilepsy. Neurology. 2016;86(10):954-62.

54. Stutterd CA, Dobyns WB, Jansen A, Mirzaa G, Leventer RJ. Polymicrogyria Overview. In: Adam MP, Ardinger HH, Pagon RA, Wallace SE, Bean LJH, Stephens K, et al., editors. GeneReviews(®). Seattle (WA): University of Washington, Seattle

Copyright © 1993-2020, University of Washington, Seattle. GeneReviews is a registered trademark of the University of Washington, Seattle. All rights reserved.; 1993.

55. Swango KL, Demirkol M, Hüner G, Pronicka E, Sykut-Cegielska J, Schulze A, et al. Partial biotinidase deficiency is usually due to the D444H mutation in the biotinidase gene. Human genetics. 1998;102(5):571-5.

56. Taft RJ, Vanderver A, Leventer RJ, Damiani SA, Simons C, Grimmond SM, et al. Mutations in DARS cause hypomyelination with brain stem and spinal cord involvement and leg spasticity. American journal of human genetics. 2013;92(5):774-80.

57. Tester DJ, Will ML, Haglund CM, Ackerman MJ. Compendium of cardiac channel mutations in 541 consecutive unrelated patients referred for long QT syndrome genetic testing. Heart rhythm. 2005;2(5):507-17.

58. Uchino T, Haraguchi Y, Aparicio JM, Mizutani N, Higashikawa M, Naitoh H, et al. Three novel mutations in the liver-type arginase gene in three unrelated Japanese patients with argininemia. American journal of human genetics. 1992;51(6):1406-12.

59. Vargas-Poussou R, Feldmann D, Vollmer M, Konrad M, Kelly L, van den Heuvel LP, et al. Novel molecular variants of the Na-K-2Cl cotransporter gene are responsible for antenatal Bartter syndrome. American journal of human genetics. 1998;62(6):1332-40.

60. Volikos E, Robinson J, Aittomäki K, Mecklin JP, Järvinen H, Westerman AM, et al. LKB1 exonic and whole gene deletions are a common cause of Peutz-Jeghers syndrome. Journal of medical genetics. 2006;43(5):e18.

61. Wiltshire E, Davidzon G, DiMauro S, Akman HO, Sadleir L, Haas L, et al. Juvenile Alpers disease. Archives of neurology. 2008;65(1):121-4.

62. Wolf B. Biotinidase Deficiency. In: Adam MP, Ardinger HH, Pagon RA, Wallace SE, Bean LJH, Stephens K, et al., editors. GeneReviews(®). Seattle (WA): University of Washington, Seattle

Copyright © 1993-2020, University of Washington, Seattle. GeneReviews is a registered trademark of the University of Washington, Seattle. All rights reserved.; 1993.

63. Sun A, Crombez EA, Wong D. Arginase Deficiency. In: Adam MP, Ardinger HH, Pagon RA, Wallace SE, Bean LJH, Stephens K, et al., editors. GeneReviews(®). Seattle (WA): University of Washington, Seattle

Copyright © 1993-2020, University of Washington, Seattle. GeneReviews is a registered trademark of the University of Washington, Seattle. All rights reserved.; 1993.

64. Zaki MS, Bhat G, Sultan T, Issa M, Jung HJ, Dikoglu E, et al. PYCR2 Mutations cause a lethal syndrome of microcephaly and failure to thrive. Annals of neurology. 2016;80(1):59-70.
