# Supplementary figures and images for: Urine creatine metabolite panel as a screening test in neurodevelopmental disorders
Source: Orphanet J Rare Dis. 2020 Dec 2;15:339. doi: 10.1186/s13023-020-01617-z (PMC7709238; doi:10.1186/s13023-020-01617-z)

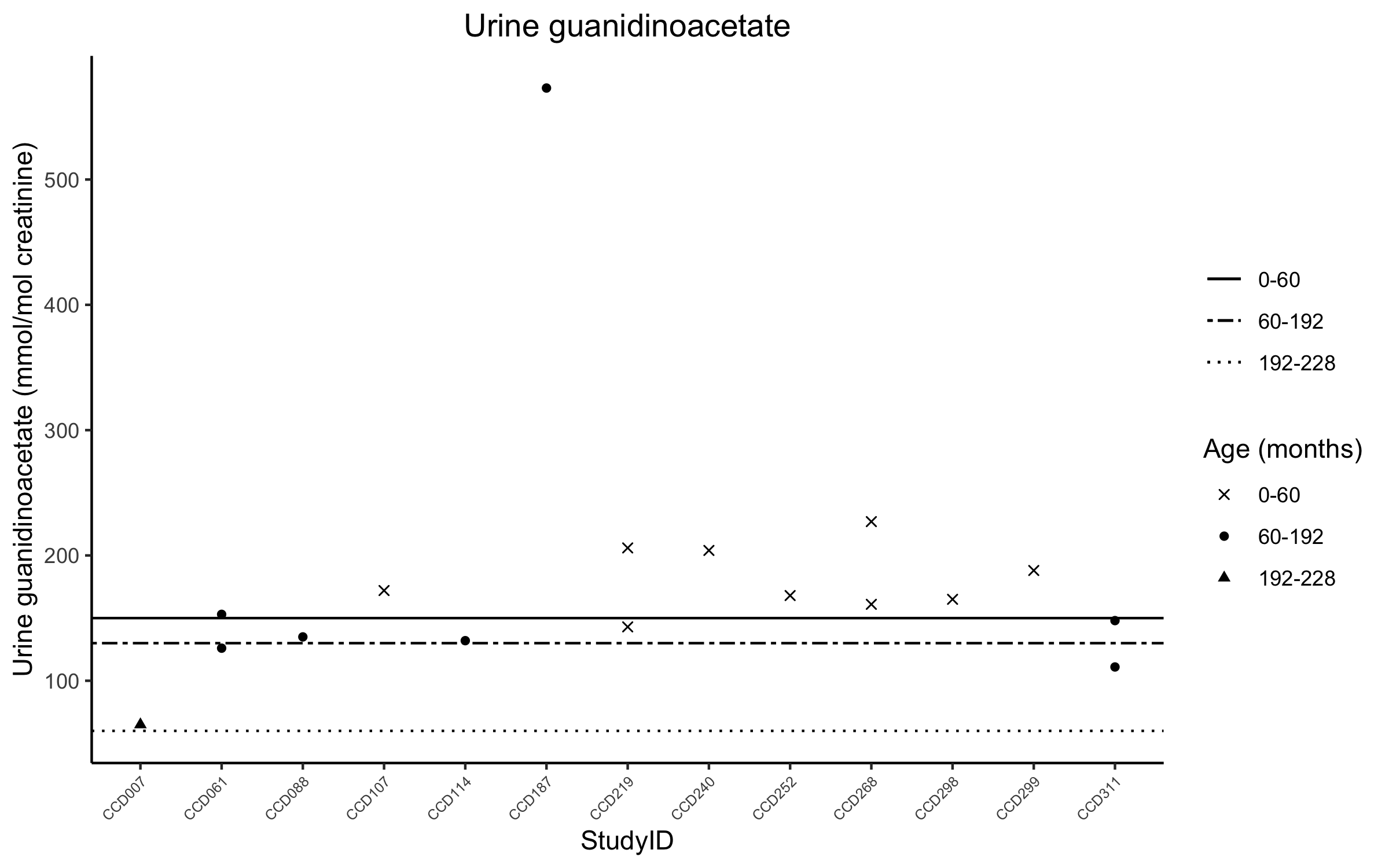

Supplement: Supplementary file 2 — Additional file 2. Elevated urine guanidinoacetate levels in patients, who had normal brain MRS or molecular genetic investigations or both investigations, are depicted in Supplemental Figure 1. Red line shows upper limit of reference range. [file 13023_2020_1617_MOESM2_ESM.tiff]

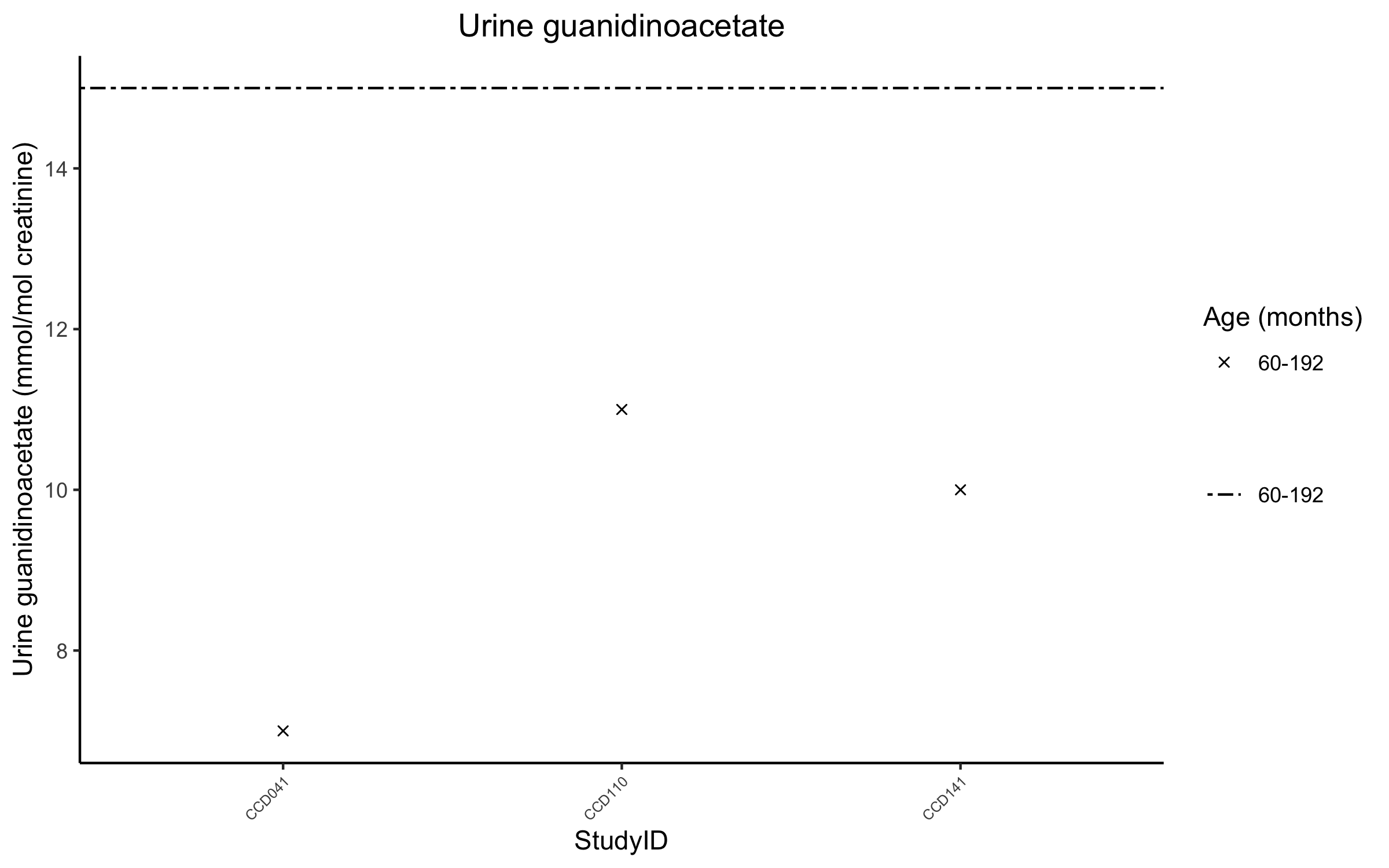

Supplement: Supplementary file 3 — Additional file 3. Low urine guanidinoacetate levels in patients, who had normal brain MRS or molecular genetic investigations or both investigations, are depicted in Supplemental Figure 2. Red line shows lowest limit of reference range. [file 13023_2020_1617_MOESM3_ESM.tiff]

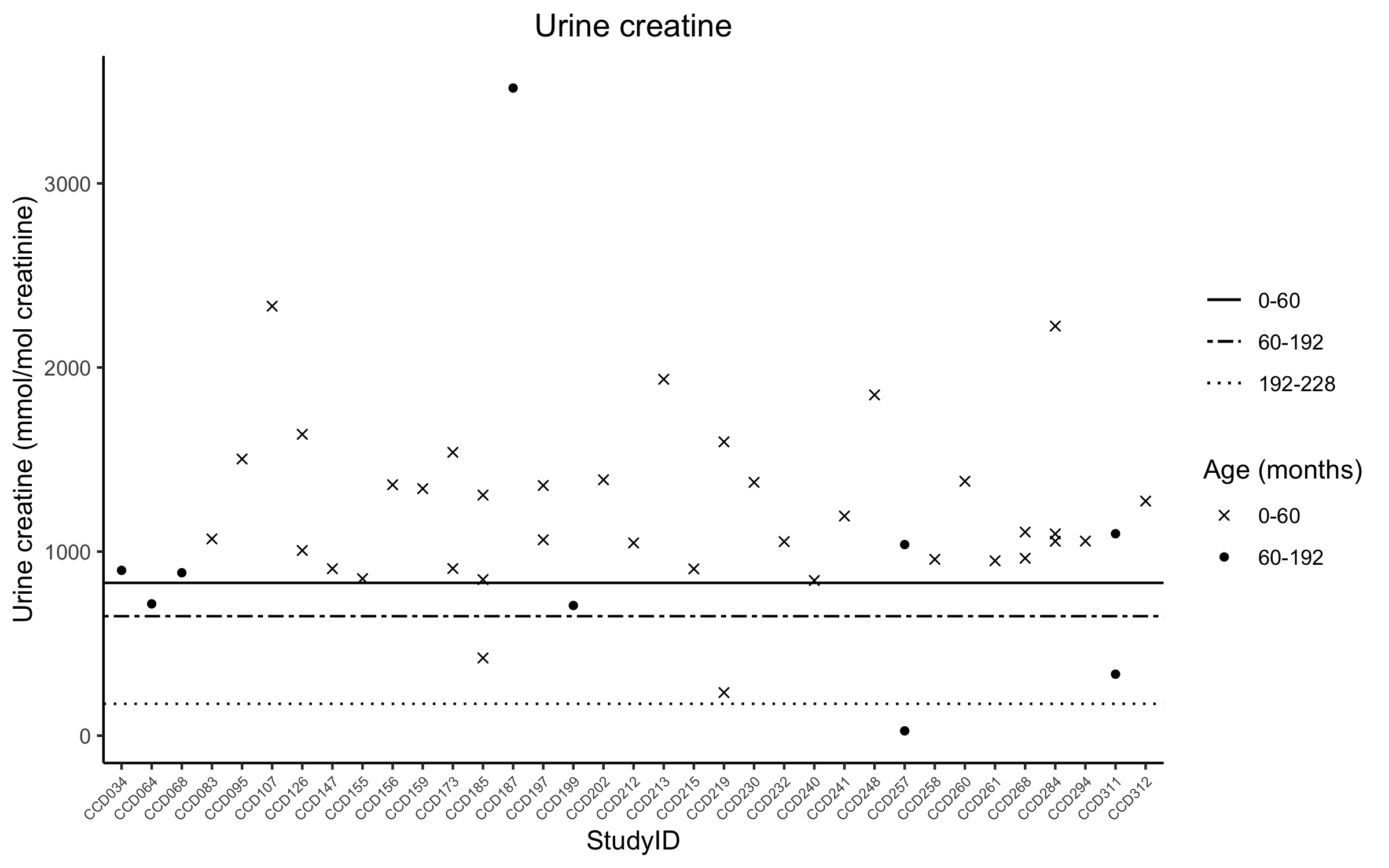

Supplement: Supplementary file 4 — Additional file 4. Elevated urine creatine in patients, who had normal brain MRS or molecular genetic investigations or both investigations, are depicted in Supplemental Figure 3. Red line shows upper limit of reference range. [file 13023_2020_1617_MOESM4_ESM.tiff]
